# Acute occlusion of the celiac axis and its branches with perforation of gastric fundus and splenic infarction, findings on spiral computed tomography: a case report

**DOI:** 10.1186/1757-1626-3-82

**Published:** 2010-03-22

**Authors:** Nikolaos L Kelekis, Evangelos Athanassiou, Dimitra Loggitsi, Rebecca Moisidou, George Tzovaras, Ioannis Fezoulidis

**Affiliations:** 1 2nd Department of Radiology, National and Kapodistrian University of Athens, General University Hospital Attikon, Rimini 1str, 12462, Athens, Greece; 2 Department of Surgery, Medical School, University of Thessalia, Papakiriazi 22 str, 41222 Larissa, Greece; 3 Department of Radiology, University of Thessalia, Medical School, University of Thessalia, Papakiriazi 22 str, 41222 Larissa, Greece

## Abstract

We present the contrast-enhanced spiral CT findings in a case of acute celiac artery occlusion with gastric perforation and total splenic infarction. Spiral CT depicted thrombus in the celiac axis and its branches, stenosis of the superior mesenteric artery, splenic infarction and lack of enhancement of the gastric wall with a large necrotic gap. Spiral CT enabled prompt diagnosis and therapy in this rare condition in a patient with suspicion of acute mesenteric ischemia.

## Introduction

Acute celiac axis occlusion is an infrequent event. Acute or chronic mesenteric ischemia occurs more commonly from superior mesenteric artery (SMA) occlusion and may result in small bowel necrosis. It may be assessed with contrast-enhanced spiral CT, and the use of multi-detector row CT scanners increases diagnostic accuracy [1]. We report a rare case of acute celiac axis occlusion and stenosis of SMA with gastric perforation and total splenic infarction diagnosed on contrast-enhanced spiral CT.

## Case presentation

An 80-year-old woman was admitted to our hospital with two days' history of dyspnea and deteriorating left sided abdominal pain. Past medical history was notable for diabetes mellitus, hypertension and coronary artery disease. She had been on digitalis, gliclazide, felodipine and isosorbide mononitrate for years. On physical examination, she was overweight, in poor general condition, with overt dyspnea, tachypnea, pallor, and dry skin. Abdomen was distended with absent bowel sounds, muscular rigidity and deep tenderness on palpation, worse on the left upper and lower quadrants. Chest examination revealed adequate left lower lung excursion but diminished breath sounds. Both legs were oedematous with normal peripheral arterial pulses.

Admission laboratory tests on were remarkable for: WBC 18600 (83.6% granulocytes), glucose 321 mg/dL (normal range 65-110 mg/dL), LDH 556 u/L (normal range 120-230), Na 130 mmol/L, K 3.1 mmol/L. ECG showed atrial fibrillation and signs of chronic digitalis therapy. Blood gases were: pO_2 _51 mmHg, pCO_2 _33.9 mmHg, PH 7.51, SO2 88.9%.

**Figure 1:**
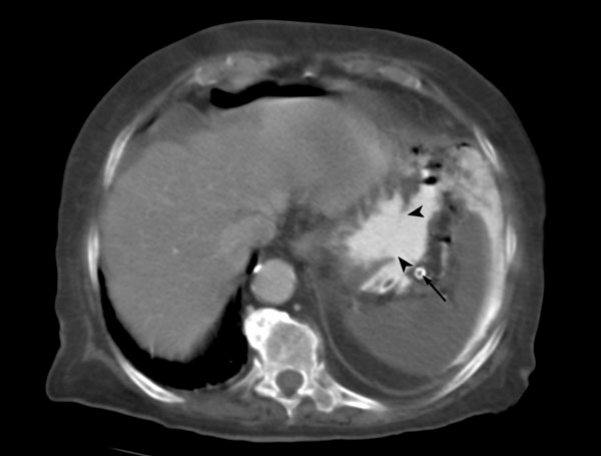
**Contrast-enhanced spiral CT shows a large gap at the stomach wall (arrowheads) with lack of enhancement and spillage of oral contrast in the peritoneal cavity**. The spleen is uniformly hypodense with thrombus (arrow) in the lumen of the calcified splenic artery. A moderate amount of perihepatic fluid and free peritoneal air are also present.

**Figure 2:**
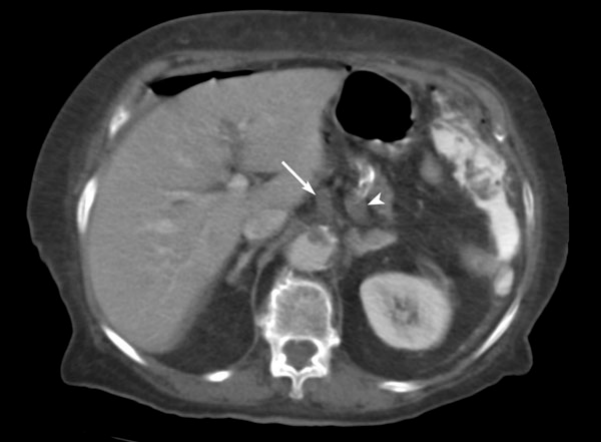
**Contrast-enhanced spiral CT shows thrombus in the trunk of the celiac artery (arrow) protruding in the lumen of the abdominal aorta, and extending into the splenic artery (arrowhead)**.

**Figure 3:**
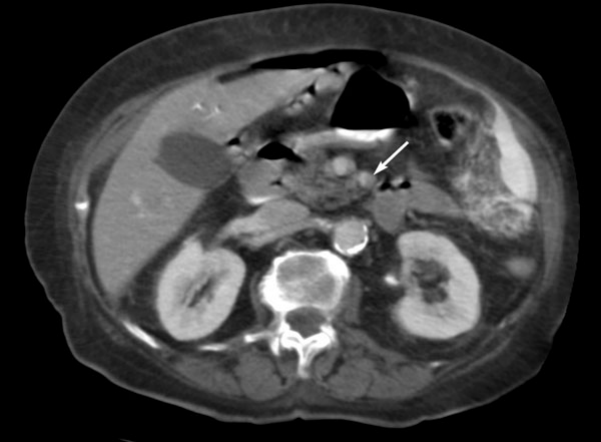
**Contrast-enhanced spiral CT at a more caudal level than on Figure 2 demonstrates significant stenosis of the proximal part of the superior mesenteric artery (arrow)**.

A CT scan of the abdomen was performed on a single-detector-row spiral scanner (SR-5000, Philips Medical Systems, Netherlands) after oral contrast administration (Gastrografin, Schering, Germany) and intravenous injection of 150 ml of 300 mgI/ml non-ionic contrast medium (Imagopaque, Amersham Health, Norway), administered with a power injector and followed by 50 ml of normal saline flush. Contrast enhanced scans were obtained during the hepatic portal dominant phase in the upper abdomen and venous phase in the pelvis with beam collimation of 5 mm, table increment of 10 mm, and reconstruction interval of 4 mm. Spiral CT showed necrosis of the stomach wall with absence of enhancement and presence of a large gap at the left lateral surface with leakage of contrast medium into the peritoneal cavity (Figure 1). The spleen was hypodense with uniform lack of enhancement. There was also free intraperitoneal air present and a moderate amount of peritoneal fluid on the right. Hypodense thrombus was present in the trunk of the celiac artery protruding in the lumen of the abdominal aorta (Figure 2), and extending into the splenic artery, common hepatic and left gastric arteries. Atherosclerotic stenoses were present at the origin and the proximal part of the SMA (Figure 3).

Following initial resuscitation, the patient underwent midline laparotomy in the operating room. Free bile-stained peritoneal fluid and a 7 cm perforation of the gastric fundus were found, the spleen was infarcted and dark coloured, while the remaining viscera were normal. The stomach was repaired using running absorbable suture in two layers, after trimming the edges of the perforation and splenectomy was performed. The patient had good immediate postoperative recovery but died of cardiopulmonary complications on the 4^th ^postoperative day.

## Discussion

Acute celiac artery obstruction occurs as a result of atherosclerosis, aneurysm, dissection, embolisation of intracardiac thrombi in atrial fibrillation, coagulation disorders caused by HIV infection, external arterial compression by the median arcuate ligament [2-4], or as complication of procedures such as Nissen fundoplication and chemoembolisation [5,6]. Long-term ergotamine abuse or splenic artery occlusion may cause ischemic necrosis of the gastric wall or upper gastrointestinal bleeding [7,8].

Ischemic necrosis occurs more frequently in organs supplied by end arteries. Gastric ischemia is uncommon as the stomach has a rich blood supply from branches of the celiac axis, as well as from SMA collaterals. A recent prospective study reported two cases of combined celiac and inferior artery obstruction with acute mesenteric ischemia without, however, signs of gastric ischemia [1]. The perforation in our case occurred in the gastric fundus, an area supplied mainly by the left gastroepiploic and short gastric arteries. There was extensive thrombus in the celiac artery and its branches with coexisting stenoses of the origin and proximal SMA, which may have contributed to the development of irreversible gastric ischemia and necrosis. Digitalis may have been an additional aggravating factor, as ischemic necrosis of gastric wall has been reported after digitalis intoxication [9], while digitalis therapy has been known to cause pooling of blood in the splanchnic vessels and spasm of portal venules in the presence of hypokalemia [10]. An interesting finding in our case was the absence of ischemic changes in the small bowel.

In splenic infarction occurring in various clinical conditions [11], pain usually resolves gradually without clinical sequelae and surgery is required only when complications arise [12]. In our case, surgery and simple closure of the stomach defect was performed instead of major gastric resection due to the presence of gastric perforation and the patient's serious clinical condition [7].

Caution should be exercised when interpreting focal stomach wall discontinuity on CT studies, as it may also be encountered in perforated gastric ulcers [1]; in our case, however, signs of ischemia, such as lack of enhancement and intraarterial thrombus were present. Lack of enhancement of ischemic wall, perforation and solid organ infarction have been identified as bad prognostic signs in acute mesenteric ischemia [1]. All these signs were present in our case, where the patient died despite prompt diagnosis and surgical intervention.

In conclusion, contrast enhanced spiral CT may accurately depict acute celiac artery occlusion and its sequelae. Accompanying gastric ischemia is very uncommon, radiologists should, however, be aware of its possibility and imaging findings, and evaluate carefully stomach wall enhancement in patients scanned with suspicion of acute mesenteric ischemia.

## Abbreviations

CT: computed tomography; EBC: erythrocyte blood cell count; ECG: electrocardiogram; LDH: lactic dehydrogenase; SMA: superior mesenteric artery; WBC: white blood cell count.

## Consent

Consent for publication was not gained before the patient died. The patient's family gave their oral consent for publication. We believe that the patient cannot be identified from this case, and that there is no reason to think that the patient or their family would have objected to publication.

## Competing interests

The authors declare that they have no competing interests.

## Authors' contributions

NLK made substantial contributions to analysis and interpretation of data and was a major contributor in writing the manuscript. EA made substantial contributions to conception and design and was a major contributor in writing the manuscript. DL made substantial contributions to acquisition, analysis and interpretation of data, as well as literature search. RM made substantial contributions to acquisition, analysis and interpretation of data. GT made substantial contributions to acquisition, analysis and interpretation of data. IF revised the manuscript critically for important intellectual content. All authors read and approved the final manuscript.
